# Goeckerman Therapy of Psoriasis: Genotoxicity, Dietary Micronutrients, Homocysteine, and *MTHFR* Gene Polymorphisms

**DOI:** 10.3390/ijms20081908

**Published:** 2019-04-17

**Authors:** Martin Beranek, Andrea Malkova, Zdenek Fiala, Jan Kremlacek, Kvetoslava Hamakova, Lenka Zaloudkova, Pavel Borsky, Tomas Adamus, Vladimir Palicka, Lenka Borska

**Affiliations:** 1 Institute of Clinical Biochemistry and Diagnostics, University Hospital Hradec Kralove and Faculty of Medicine in Hradec Kralove, Charles University, 50005 Hradec Kralove, Czech Republic; lenka.zaloudkova@fnhk.cz (L.Z.); palicka@lfhk.cuni.cz (V.P.); 2 Department of Biochemical Sciences, Faculty of Pharmacy in Hradec Kralove, Charles University, 50005 Hradec Kralove, Czech Republic; 3 Institute of Hygiene and Preventive Medicine, Faculty of Medicine in Hradec Kralove, Charles University, 50003 Hradec Kralove, Czech Republic; malka8ar@lfhk.cuni.cz (A.M.); fiala@lfhk.cuni.cz (Z.F.); borskyp@lfhk.cuni.cz (P.B.); 4 Institute of Pathological Physiology, Faculty of Medicine in Hradec Kralove, Charles University, 50003 Hradec Kralove, Czech Republic; kremlack@lfhk.cuni.cz (J.K.); borka@lfhk.cuni.cz (L.B.); 5 Clinic of Dermatology and Venereology, University Hospital Hradec Kralove, 50005 Hradec Kralove, Czech Republic; kveta.hamakova@fnhk.cz; 6 Department of Biomedical Sciences, Faculty of Medicine, University of Ostrava, 70300 Ostrava, Czech Republic; Tomas.Adamus@osu.cz

**Keywords:** psoriasis, Goeckerman therapy, genotoxicity, homocysteine, vitamin B12, folic acid

## Abstract

Goeckerman therapy (GT) of psoriasis vulgaris is based on the application of crude coal tar and ultraviolet radiation. We investigated DNA damage by the number of micronucleated binucleated cells (MNBC) in lymphocytes, serum homocysteine, vitamin B12, folic acid, and two polymorphisms (C677T and A1298C) in the *MTHFR* gene in 35 patients with exacerbated psoriasis vulgaris classified according to the psoriasis area and severity index (PASI) score and treated by GT. The median of PASI score decreased from nineteen to five, and MNBC increased from 10 to 18‰ after GT (*p* < 0.001 in both cases). Correlations of MNBC with homocysteine (Spearman’s rho = 0.420, *p* = 0.012) and vitamin B12 (rho = −0.389, *p* = 0.021) before the therapy were observed. Hyperhomocysteinemia was an independent predictor of genotoxicity (OR 9.91; 95% CI, 2.09–55.67; *p* = 0.003). Homocysteine was higher in females than in males (13 vs. 12 µmol/L, *p* = 0.045). In contrast, vitamin B12 levels in the females were lower than in the males (160 vs. 192 pmol/L, *p* = 0.047). Vitamin B12 in the females were negatively influenced by smoking status (160 pmol/L in smokers vs. 192 pmol/L in non-smokers, *p* = 0.025). A significantly higher MNBC was found in CC homozygous patients (A1298C polymorphism) than in AC heterozygotes (32 vs. 16‰, *p* = 0.005) and AA homozygotes (32 vs. 18‰, *p* = 0.036). Our data showed that homocysteine participates in the pathogenesis of psoriasis. Its serum levels correlated with MNBC and allowed the prediction of DNA damage to appear within GT. Both micronutrients status and homocysteine metabolic pathway contribute to the genotoxicity of GT.

## 1. Introduction

Psoriasis vulgaris is a chronic skin disease of multifactorial origin, which affects 1–3% of the worldwide population [1]. The majority of patients with mild, mild-to-moderate psoriasis and part of severe cases are treated with topical agents involving corticosteroids, vitamin D3 analogs, retinoids, keratolytics, anthralin, calcineurin inhibitors, and pharmaceutical crude coal tar (CCT) ointment [2].

In dermatological practice, the application of CCT is often combined with ultraviolet radiation (UVR), the procedure known as the Goeckerman therapy (GT) [3]. The therapeutic effect of UVR consists of the induction of chemical changes in chromosomal DNA that slow down the hyperproliferation of skin cells in psoriatic lesions [4]. The therapeutic impact of CCT, which contains variable amounts of genotoxic and immunotoxic polycyclic aromatic hydrocarbons (PAHs), seems to be very complex. Apart from keratolytic, antibacterial, anti-inflammatory, antipruritic, and photosensitizing effects, CCT suppresses DNA synthesis, which together with UVR contributes to the slower proliferation of sick keratinocytes [5].

The GT is considered as a relatively cheap, safe, and effective therapeutic approach with long-term remission [6]. On the other hand, it is known that combined exposure to UVR and PAHs generates reactive oxygen species, irreversible DNA adducts, double-strand DNA breaks, and structurally aberrated chromosomes [7,8,9].

Dietary micronutrients, including vitamin B12 and folic acid, maintain genetic stability in a cell. Their deficiencies result in the reduction of S-adenosyl methionine synthesis, hyperhomocysteinemia, altered DNA methylation, and elevated DNA damage [10]. Lower serum levels of vitamin B12 and folic acid and higher levels of homocysteine in psoriasis patients than in healthy subjects were described [11]. Several authors found significant associations of homocysteine with psoriasis duration and severity [12,13], though others did not support their findings [14].

Methylenetetrahydrofolate reductase (MTHFR) is an enzyme converting 5,10-methylenetetrahydrofolate to 5-methyltetrahydrofolate, which provides methyl groups for the remethylation of homocysteine to methionine. Several genetic polymorphisms in the *MTHFR* gene associate with reduced MTHFR activity, decreased vitamin B12 and folic acid, and increased homocysteine levels in the blood. In psoriatic patients, the relationship between C677T polymorphism and the severity of the disease was observed [15]; however, the data are still inconsistent, and other authors did not confirm this association [16].

In this study, we aimed to investigate the genotoxicity (via analysis of micronuclei), serum levels of homocysteine, vitamin B12 and folic acid, and C677T (rs1801133) and A1298C (rs1801131) polymorphisms in the *MTHFR* gene in patients with exacerbated psoriasis vulgaris treated by Goeckerman therapy.

## 2. Results

The psoriasis area and severity index score in the patients ranged from 10 to 42 (median value 19, interquartile range 14–22). After the therapy, the median PASI score decreased to five (4–7, *p* < 0.001), Table 1. Twenty patients had a moderate form of plaque psoriasis (median PASI score 15, 13–16), and fifteen patients had severe plaque psoriasis vulgaris (median PASI score 23, 21–29). Between the patients with moderate and severe psoriasis, we only found insignificant differences in age distribution (34–62 and 35–56 years, *p* = 0.467) and body mass index (BMI) (median, 28.5 and 28.4 kg/m^2^, *p* = 0.701). Also, differences in the efficiency of GT between patients with moderate and severe psoriasis were insignificant (69.9 vs. 73.9%, *p* = 0.303).

**Figure 1:**
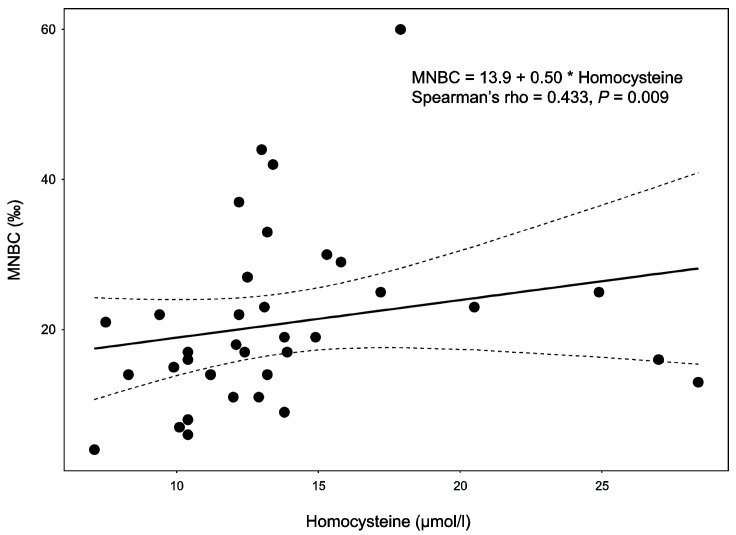
Relationship between micronucleated binucleated cells (MNBC) and serum levels of homocysteine after Goeckerman therapy. The solid line depicts linear regression fit with the 95% confidence boundaries plotted as dashed curves.

Table 1 shows that MNBC in lymphocytes, the evaluated parameter of genotoxicity after GT, significantly increased to the baseline values (18 vs. 10‰, *p* < 0.001). Twenty-four patients (70%) revealed higher MNBC than the reference range 3–16‰ [17]. No significant association of MNBC with age, gender, BMI, smoking status, disease severity, length of irradiation, or duration of treatment was apparent. A positive correlation of MNBC with homocysteine was found both before GT (Spearman’s rho = 0.420, *p* = 0.012) and after GT (rho = 0.433, *p* = 0.009, Figure 1), respectively. Also, MNBC was negatively associated with vitamin B12 before GT (rho = -0.389, *p* = 0.021).

As shown in Table 1, insignificant differences in serum homocysteine, vitamin B12, and folic acid were obtained when the data before and after the treatment were compared (12 vs. 13 µmol/L, *p* = 1.000; 183 vs. 178 pmol/L, *p* = 0.713; 12.0 vs. 11.6 nmol/L, *p* = 0.082, respectively). Similarly, we did not find any significant differences between moderate and severe cases in homocysteine (12 vs. 13 µmol/L, *p* = 0.526), vitamin B12 (192 vs. 175 pmol/L, *p* = 0.474), and folic acid concentrations (11.9 vs. 13.2 nmol/L, *p* = 0.627).

In sixteen patients (46%), the baseline serum homocysteine was higher than the reference range (5–12 µmol/L), and only four patients (11%) showed the lower levels of homocysteine than the mean range (8.5 µmol/L). Additionally, hyperhomocysteinemia determined before GT was found to be an independent predictor of a higher risk of genotoxicity: odds ratio (OR), 4.29; 95% CI, 1.06–19.11; *p* = 0.040. The homocysteine measured after GT provided even a noticeably stronger predictive value: OR, 9.91; 95% CI, 2.09–55.67; *p* = 0.003.

All the patients had vitamin B12 levels lower than the value of the first quartile (314 pmol/L) of the reference range (133–675 pmol/L), and six of them (17%) suffered from vitamin B12 deficiency. The serum concentration of homocysteine negatively correlated with folic acid (rho = −0.364, *p* = 0.031 before GT). A total of 34 patients (97%) had folic acid levels lower than the first quartile (25.0 nmol/L) of the appropriate reference range (9.1–56.7 nmol/L), and in ten of them (29%), folic acid hypovitaminosis was determined. 

When gender differences were evaluated, the level of homocysteine was found significantly higher in the psoriatic females than in the males before (13 vs. 12 µmol/L, *p* = 0.045) and after GT (14 vs. 12 µmol/L, *p* = 0.016). In contrast, vitamin B12 levels in the females were significantly lower than in the males before GT (160 vs. 192 pmol/L, *p* = 0.047) and after GT (160 vs. 189 pmol/L, *p* = 0.010). Additionally, the baseline values of vitamin B12 in the females were negatively influenced by smoking status (160 pmol/L in smokers vs. 192 pmol/L in non-smokers, *p* = 0.025). MNBC and folic acid did not differ significantly before GT (11 vs. 8‰, *p* = 0.248 and 11.2 vs. 12.0 nmol/L, *p* = 0.179, respectively), as well as after GT (19 vs. 17‰, *p* = 0.689 and 11.0 vs. 11.9 nmol/L, *p* = 0.147, respectively).

The distribution of CC/CT/TT genotypes of *MTHFR* C677T polymorphism (rs1801133) and AA/AC/CC distribution of A1298C polymorphism (rs1801131) in the experimental and the control groups conformed to the Hardy-Weinberg equilibrium (*p* values 0.390 and 0.070 for C677T, 0.224 and 0.390 for A1298C, respectively). The frequencies of CC, CT, TT genotypes in C677T polymorphism were 34.3%, 54.3%, 11.4% in the case group and 55.0%, 31.7%, 13.3% in the controls. The frequency of the TT homozygous patients and that of the controls did not reach a significant difference (*p* = 0.087). The frequencies of AA, AC, CC genotypes in A1298C polymorphism were 57.1%, 31.4%, and 11.4% in the cases and 41.7%, 41.7%, and 16.6% in the controls, respectively. No significant difference in the minor CC genotype frequencies was observed (*p* = 0.343) between the cases and controls.

After GT, a significantly higher frequency of MNBC was found in the CC homozygous patients (A1298C polymorphism) than in the AC heterozygotes (32 vs. 16‰, *p* = 0.005) and the AA homozygotes (32 vs. 18‰, *p* = 0.036). None of the investigated polymorphisms significantly influenced serum levels of homocysteine, vitamin B12, and folic acid, although marginally significant trends to a higher homocysteine and a lower folic acid were apparent in the T allele carriers (C677T polymorphism) when compared to the C allele homozygotes (13 vs. 10 µmol/L, *p* = 0.051 and 10.4 vs. 14.7 nmol/L, *p* = 0.063, respectively).

## 3. Discussion

Our results showed that Goeckerman therapy, combining CCT administration with UVR, is an effective way to reduce the PASI score in moderate and severe psoriasis vulgaris. To evaluate its genotoxic effect, we performed an analysis of micronuclei in the patients’ lymphocytes. This parameter, resulting from either whole chromosome loss or breakage, sensitively determines DNA damage induced by oxidative stress in inflammatory conditions [10]. To the best of our knowledge, no one has yet evaluated the genotoxic potential of GT by using this test.

After dermal exposure, CCT substances (i.e., PAHs and their active metabolites) penetrate through the layer of stratum corneum and reach the subpapillary arteriovenous plexus or go to deeper subcutal vessels [18], appear in the blood and could influence lymphocytes in the circulation. UVR is absorbed in the surface layer of the epidermis [19] and forms reactive oxygen species subsequently affecting blood lymphocytes [20], as well.

We found that the frequency of micronuclei significantly increased after the applied treatment, independently on the psoriasis severity or other clinical variable tested. Our data support the results of the previous studies [7,8,9] and definitely manifest the genotoxic effect of GT. The total number of MNBC obtained in psoriasis patients after GT was very similar to the values found in the group of the Turkish asphalt workers (mean ± SD; 21.6 ± 2.5‰) [21] but lower than in the sewage workers in Paris (38.0 ± 7.2‰) [22]. On the other hand, our results were higher than those obtained in the group of dermatological patients treated only by comparable doses of UVR (311 nm, 14.7 ± 7.7‰) [23].

Homocysteine might be involved in the pathogenesis of psoriasis [24]. Almost half of our patients suffered from hyperhomocysteinemia, which positively correlated with increasing numbers of micronucleated cells. A similar relationship was formerly observed in patients with coronary artery disease [25] and in older men [26]. We revealed that increased serum homocysteine in psoriasis patients could serve as a promising predictive marker of DNA damage after GT. Finding hyperhomocysteinemia might allow initiating appropriate dietary intervention before the treatment and minimizing its genotoxic impact. 

It is unclear, however, whether homocysteine itself induces the DNA damage or whether it is a biomarker of other events leading to DNA instability. Hyperhomocysteinemia is known to reflect vitamin B12 or folic acid deficiency. In psoriasis, reduced serum concentrations of vitamin B12 were previously reported [11], although other studies presented its normal levels [12,16]. Most of our patients had vitamin B12 within the reference range with a noticeable shift to the left, especially in the females. Their baseline data for vitamin B12 (but not for homocysteine or folate) were negatively modulated by smoking habit, albeit they were only light smokers consuming up to five cigarettes per day. In agreement with the study of Vanizor Kural et al. [11], vitamin B12 in the patients negatively correlated with homocysteine and also with MNBC. Since the status of vitamin B12 remained unchanged after the treatment, it seems that GT does not influence its serum concentrations.

Several papers reported a lower folic acid in psoriasis serum than in healthy controls, which was explained by decreased intestinal absorption and increased folate utilization in lesional keratinocytes [11,12,27]. In the present study, we observed that folic acid in the patients lied below the mean of the reference range, and ten individuals were deficient in this micronutrient. The levels of folic acid were positively associated with vitamin B12, but we did not find any correlation between folic acid and PASI score or homocysteine, despite the previous publication of such correlations [12].

Fenech et al. have suggested that minimal serum concentrations of vitamin B12 and folic acid were needed for maintenance of normal frequencies of micronuclei in lymphocytes, that is, 148 pmol/L and 9.1 nmol/L, respectively [10]. Since over 70% of our patients had vitamin B12 and folic acid higher than these limits, the elevated MNBC did not exclusively result from their dietary deficiencies before or after GT.

The next part of the study examined potential metabolic reasons for increased homocysteine and MNBC in psoriasis. We focused on the effects of two common polymorphisms in the *MTHFR* gene. The C677T polymorphism is known to modulate serum or plasma concentrations of homocysteine. In TT homozygotes, low MTHFR activity resulting in increased homocysteine and DNA damage was reported [28,29]. Psoriasis patients with the TT genotype manifested different PASI score, homocysteine, and folic acid than CC homozygous patients [15], although others did not support these findings [16,30,31]. Discrepancies between the studies might be caused by many reasons, including different sample sizes used in these studies and *MTHFR* gene variation in the ethnicities investigated. In the present study, the distribution of C677T genotypes among the patients was similar to healthy controls. In the carriers of T allele, we found only an insignificant trend to higher homocysteine and no differences in vitamin B12 and folic acid levels when compared to the CC homozygotes; however, the data were influenced by a low number of the patients.

In an effort to clarify the above discrepant results, we investigated the *MTHFR* A1298C polymorphism. The frequency of mutated C allele in psoriasis was previously found to be higher than in healthy individuals [32]. In our study, we found no significant differences between the distribution of AA, AC, and CC genotypes and the control group. Differences among the A1298C genotypes were observed neither in homocysteine nor in vitamin B12 nor in folic acid. On the other hand, we noticed a significantly higher MNBC in the CC homozygotes than in the AC and the AA genotypes. To the best of our knowledge, this study for the first time indicates a possible relationship between the *MTHFR* C1298A polymorphism and DNA damage caused by Goeckerman therapy. However, because of a limited number of our psoriasis patients, broader studies are needed to support our preliminary data. 

## 4. Materials and Methods 

### 4.1. Subjects

The experimental group consisted of 35 patients (20 females and 15 males) with exacerbated psoriasis vulgaris whose median age was 53 years, range 18–82 years. Fourteen of them were smokers and 21 nonsmokers. According to the basic characteristics of actual disease status (erythema, desquamation, and skin infiltration), the activity of disease was expressed as the psoriasis area and severity index (PASI) score. The disease with PASI score <10 was classified as a mild, 10–20 as a moderate, and >20 as severe psoriasis. 

The patients were without any treatment for at least two weeks before enrollment to the study; none of them had systemic psoriasis therapy ever applied. Their exposure history was examined using a questionnaire. The patients who had significant prior exposure to PAHs and/or artificial UVR and patients with acute infections, psoriatic arthritis, or other inflammatory diseases were excluded. Samples from 60 healthy controls (27 females and 33 males, median age 50 years, range 21–66 years) were used for *MTHFR* genotyping. Neither the patients nor the controls were treated by any drugs influencing inflammatory response. The study was conducted in accordance with the Declaration of Helsinki, and the study protocol was approved by the Ethics Committee of the Charles University Hospital in Hradec Kralove, Czech Republic (project identification code 201705183P, approved 02/05/2017). Informed written consent was obtained from each subject.

### 4.2. Goeckerman Therapy

Depending on the extent of skin lesions, 15–60% of the total body surface area was covered by dermatological ointment with 4% coal tar each day. After removing ointment residues in an oil bath, total body UVR was applied. The duration of radiation was tailored individually according to the disease status (1–15 min). The density of the used UVR reached values of 249.64 mW/cm^2^ of UV-B and 131.5 mW/cm^2^ of UV-A (Sola-Scope 2000, Solatell, Croydon, UK). The duration of the treatment was continually modified according to the treatment efficiency calculated as follows: 100*[(PASI_before_–PASI_after_)/PASI_before_] (%). The mean duration of GT was 13 days with a range of 3–23 days.

### 4.3. Analysis of Genotoxicity

Venous blood was collected into Li-heparinized tubes (Vacuette, Mundelein, IL, USA) one day before the first and immediately after the last GT procedure. Samples were stored at 4–8 °C and processed for subsequent cytogenetic analysis within 24 h after the collection. The cultures were initiated with 1 mL of blood in 7.5 mL of RPMI 1640 medium (Sigma-Aldrich, St. Louis, MO, USA) supplemented with 20% fetal bovine serum (Biosera, Kansas City, MO, USA), 2% phytohemagglutinin (Gibco, Grand Island, NY, USA), and 1% penicillin-streptomycin (Sigma-Aldrich), and incubated at 37 °C for 72 h. Cytochalasin B (5.33 µg/mL, PanReac AppliChem, Darmstadt, Germany) was added in the forty-fourth hour of incubation. At the seventy-second hour, the cells were harvested by centrifugation (200× *g*, 8 min) and re-suspended in 10 mL of pre-warmed (37 °C) 0.075 M KCl for 5 min, fixed in the first step by 10 mL of methanol:acetic acid (3:1) with 1% formaldehyde, and in the second and the third steps by 10 mL of methanol:acetic acid (3:1). Then, the cells were dropped onto chilled humid slides and left to dry overnight at room temperature. Next day, the slides were stained with 5% Giemsa (10 min) and analyzed by using a B-383PLi microscope (Optika, Ponteranica, Italy). In each sample, the number of micronucleated cells in 1000 binucleated cells (MNBC) was scored [33] and expressed in promiles. The normal reference range (3–16‰) was derived from previously published data [17].

### 4.4. Immunochemical Analysis

Samples of venous blood were collected into Vacutainer tubes (Becton Dickinson, Franklin Lakes, NJ, USA). The separated serum was stored at −70 °C until analysis. Repeated thawing and freezing of the samples were avoided. The serum levels of homocysteine were determined by using the Immulite 2000 Homocysteine kit (Siemens, Erlangen, Germany). Dynamic and reference ranges were 2–50 µmol/L and 5–12 µmol/L, respectively. The serum concentrations of folic acid and vitamin B12 were assessed by using the Access Folate kit, Beckman Coulter, Brea, CA, USA (2.3–56.2 nmol/L, 9.1–56.7 nmol/L) and the Access Vitamin B12 kit, Beckman Coulter (37–1107 pmol/L, 133–675 pmol/L) according to the manufacturer’s instructions, respectively. The samples were used without any additional dilution, and all experiments were done in duplicates.

### 4.5. DNA Analysis

Genomic DNA of the patients and controls was extracted from EDTA blood samples (Becton Dickinson) according to the manufacturer’s instructions (QIAamp Blood Mini Kit, Hilden, Germany). The analysis of both polymorphisms was performed by using GB HEMO MTHFR (C677T) and GB HEMO MTHFR (A1298C) kits (Generi Biotech, Hradec Kralove, Czech Republic). The kits are based on real-time PCR technology employing fluorescently labeled hydrolysis probes (FAM for wild-type and HEX for mutated alleles) and certified for clinical in vitro diagnostics. The amplification and detection were carried out in the Rotor-Gene 6000 (Corbett Research, Sydney, Australia) according to the instructions of the manufacturer. Each run included the analysis of control samples with known *MTHFR* genotypes and no template control samples.

### 4.6. Statistical Analysis 

The data were statistically processed with R software version 3.5.1 using the “nortest”, “compute.es”, and the “ggplot2” packages. Based on the Anderson-Darling test for the data distribution, either the parametric or nonparametric test was used to ensure the proper test sensitivity. Associations between the laboratory and the clinical parameters were evaluated by Pearson’s correlation test or Spearman’s rank test; intergroup differences were assessed using the Student’s t-test or the Wilcoxon rank-sum test for continuous variables and the Fisher’s exact test for discrete ones. The exact test was also used to estimate the consistency of the genotype frequencies in the *MTHFR* polymorphisms with Hardy-Weinberg equilibrium. The differences were considered statistically significant when the probability level (*p*) was below the alpha level of 0.05.

## 5. Conclusions

Summarizing the findings, this study confirmed that homocysteine participates in the pathogenesis of psoriasis vulgaris. Its serum levels correlated with the frequency of micronucleated cells and allowed the prediction of DNA damage to appear within Goeckerman therapy. A potential link between the *MTHFR* C1298A polymorphism and genotoxic effects of crude coal tar and/or UVR was also found. Our study, thus, showed that both micronutrients status and homocysteine metabolic pathway contribute to the genotoxicity of Goeckerman therapy in psoriasis patients. 

## Figures and Tables

**Table 1:** Values (and interquartile ranges) of the investigated parameters.

Parameter [unit]	Psoriasis Patients		Reference
	Before GT	After GT	Range
PASI score	19	5 ^&^	-
	(14–22)	(4–7)	
MNBC [‰]	10	18 ^&^	3–16
	(7–16)	(14–25)	
Folic acid [nmol/L]	12.0	11.6	9.1–56.7
	(8.5–15.6)	(8.3–14.7)	
Homocysteine [µmol/L]	12	13	5–12
	(10–15)	(10–14)	
Vitamin B12 [pmol/L]	183	178	133–675
	(155–230)	(156–200)	

Abbreviations: GT, Goeckerman therapy; PASI, psoriasis area and severity index; MNBC, micronucleated binucleated cells; ^&^ *p* < 0.001 to the value before GT.

## Abbreviations

CCT	crude coal tar
GT	Goeckerman therapy
MNBC	micronucleated binucleated cells
MTHFR	methylenetetrahydrofolate reductase
PAHs	polycyclic aromatic hydrocarbons
PASI	psoriasis area and severity index
UVR	ultraviolet radiation
